# P05.47. Yoga for musculoskeletal conditions: a Delphi survey to establish international consensus of core intervention components

**DOI:** 10.1186/1472-6882-12-S1-P407

**Published:** 2012-06-12

**Authors:** L Ward, S Stebbings, K Sherman, D Cherkin, D Baxter

**Affiliations:** 1 School of Physiotherapy, University of Otago, Dunedin, New Zealand; 2 School of Medicine, University of Otago, Dunedin, New Zealand; 3 Group Health Research Institute, Seattle, USA

## Purpose

To develop and define a core set of key components of a yoga intervention protocol for musculoskeletal conditions, through consensus of an international panel of researchers and yoga consultants.

## Methods

Recruitment will comprise two phases. In the primary phase, individuals identified through a systematic review of the literature of yoga for musculoskeletal conditions will be invited to participate. In the secondary phase, a snowball technique will allow primary phase participants to recommend other researchers to contribute to the Delphi process. Inclusion criteria are involvement in the conception, design, conduct, teaching or analysis of randomised controlled trials or pilot interventions of yoga for musculoskeletal conditions. The Delphi process is anticipated to take three rounds, conducted via electronic surveys. The round 1 survey (beginning January 2012) will consist of one open-ended question asking suggestions for the core components of a yoga intervention protocol for musculoskeletal conditions. Rounds 2 and 3 surveys will consist of close-ended questions based on the results of the previous round. Responses are anonymous.

## Results

A steering committee will oversee survey development and analysis of results. Round 1 data will be analysed qualitatively using thematic analysis. Items generated will form the questionnaire for round 2. Questionnaire items from rounds 2 and 3 will be analysed quantitatively. Pre-determined consensus levels set by the steering committee will determine whether items are included in the subsequent rounds or discarded.

## Conclusion

The core set of key components generated by the Delphi panelists will address the current heterogeneity of content and delivery of complex yoga interventions, by providing a reference tool for best practice in protocol design. Promoting such standardisation will enable comparison and replication of yoga research internationally, while retaining the flexibility to adapt the protocol to different musculoskeletal conditions and styles of yoga.

